# Pediatric Emergency Medicine Ultrasound Fellowship Programs

**DOI:** 10.24908/pocus.v9i1.17372

**Published:** 2024-04-22

**Authors:** Sigmund J. Kharasch, Matthew Moake, Antonio Riera

**Affiliations:** 1 Massachusetts General Hospital Boston, MA USA; 2 Harvard Medical School Boston, MA USA; 3 Yale-New Haven Children's Hospital New Haven, CT USA; 4 Yale School of Medicine New Haven, CT USA; 5 Medical University of South Carolina Charleston, SC USA; 6 Shawn Jenkins Children's Hospital Charleston, NC USA

**Keywords:** Pediatric Emergency Medicine, Point of Care Ultrasound, fellowships, bedside ultrasound

## Abstract

Point of care ultrasound (POCUS) has undergone important growth in the field of Pediatric Emergency Medicine (PEM) in the last 14 years and is recognized as a critical diagnostic tool in the care of ill and injured children. The first PEM POCUS fellowship was established in 2010. Now, there are currently 30 ultrasound fellowships that offer training to PEM physicians. In 2014, 46 PEM POCUS leaders established the P2 (PEM POCUS) Network (www.P2network.org). This serves as a platform for sharing expertise, building research collaborations, and offering mentorship in the use of POCUS in PEM. In 2019, a multinational group of experts in PEM POCUS published the first consensus guidelines for prioritizing core applications of POCUS, which are fundamental to PEM fellowship training 1. In 2022, the international research priorities for PEM POCUS were published 2. In the same year, the development of a consensus-based definition of focused assessment with sonography for trauma (FAST) in children was established 3.

## Letter

Since the first publication of pediatric emergency medicine ultrasound programs in 2021 4, peer-reviewed educational resources have continued to expand through the P2 Network (www.P2network.org). These include a narrated core content lecture series incorporating the 2019 consensus POCUS applications, a narrated lecture series by pediatric POCUS experts covering advanced topics, and recordings from the Pediatric Emergency POCUS Educational Collaborative (PEPEC) presentations by pediatric POCUS leaders across the globe. The P2Network also collaborates with the World Interactive Network Focused On Critical UltraSound (WINFOCUS) to provide pediatric POCUS educational content, and is developing an online training platform. The project vision is to provide a globally accessible platform, translated into multiple languages, that will enable pediatricians to receive training and certification in pediatric POCUS through interactive and up-to-date teaching methods. 

 The following listing provides concise and essential information about each of the Pediatric Emergency Medicine Ultrasound fellowships in the United States, Canada, and United Kingdom (Table 1). The list was compiled from the Society of Clinical Ultrasound Fellowships (SCUF) and updated with input from the program leadership. Changes in the status of ultrasound programs as free-standing PEM POCUS programs or joint PEM/EM programs were similarly updated. Additional sources of information include the 2023 National Resident Matching Program, Match Participant List, and the P2Network membership. 

**Table 1:** Pediatric Emergency Medicine Ultrasound Fellowship Program Listing for the United States, Canada, and United Kingdom in 2024.

**Program**	**Program Director**	**PD E-mail**	**Fellowship Director**	**FD E-mail**	**Joint PEM/EM Fellowship**	**Website**
Baylor College of Medicine/ Texas Children’s Hospital, Houston, TX	Kiyetta Alade, MD	alade@bmc.edu	Stephanie Leung, MD	pempocushtx@gmail.com	No	https://www.bcm.edu/departments/pediatrics/divisions-and-centers/emergency-medicine/education/pediatric-point-of-care-ultrasound-fellowship
Bellevue Hospital/NYU Medical Center New York, NY	Maya Lin, MD	maya.lin@nyulangone.org	Maya Lin, MD	maya.lin@nyulangone.org	Yes	https://www.eusfellowships.com/programs/details?id=78
Brown University, Hasbro Children’s Hospital, Providence, RI	Erika Constantine, MD	erika_constantine@brown.edu	Almaz Dessie, MD	almaz@brown.edu	No	https://www.brownpem.org/fellowship
Children’s Hospital Los Angeles, Los Angeles, CA	Marsha Elkhunovich, MD	melkhunovich@chla.usc.edu	Dana Sajed, MD	dsajed@gmail.com	Yes	https://eusfellowships.com/program-extra/?program=72
Children’s Hospital of Michigan, Detroit, MI	Jennifer Noble, MD	jnoble@med.wayne.edu	Jennifer Noble, MD	jnoble@med.wayne.edu	No	https://www.eusfellowships.com/programs/details?id=202
Children’s Hospital of Eastern Ontario, Ottawa, Canada	Allan Shefrin, MD	ashefrin@cheo.on.ca	Kate Fathi, MD	kfathi@cheo.on.ca	No	https://www.uottawa.ca/faculty-medicine/postgraduate-medical-education/fellowship-areas-focused-competence/programs
Children’s Hospital of Philadelphia, Philadelphia, PA	Aaron Chen, MD	chena2@email.chop.edu	Rachel Rempell, MD	rempellr@email.chop.edu	No	https://www.eusfellowships.com/programs/details?id=109
Children’s Minnesota, Minneapolis, MN	Valerie Whitcomb, MD	valerie.whitcomb@childrensmn.org	Valerie Whitcomb, MD	valerie.whitcomb@childrensmn.org	No	https://www.eusfellowships.com/programs/details?id=198
Children’s National Medical Center, Washington, DC	Rosemary Thomas-Mohtat, MD	rthomasm@ cnmc.org	Simone Lawson,MD	sllawson@childrensnational.org	No	https://www.eusfellowships.com/programs/details?id=120
Cohen Children's Medical Center, New Hyde Park, NY	David Teng, MD	dteng@northwell.edu	Matthew Kusulas, MD	mkusulas@northwell.edu	No	https://www.eusfellowships.com/programs/details?id=110
Dalhousie University EM, Saint John Regional Hospital, Saint John, NB & IWK Health, Halifax, NS, Canada	David Lewis MBBS Kirstin Weerdenburg MD	david.lewis@ dal.ca kirstin.weerdenburg@iwk.nshealth.ca	David Lewis MBBS Kirstin Weerdenburg MD	david.lewis@dal.ca kirstin.weerdenburg@iwk.nshealth.ca	Yes	https://sjrhem.ca/programs/ultrasound/dalhousie-dem-sjrhem-iwk-pocus-fellowship-elective/
Denver Health, Denver CO	Amanda Greene Toney, MD	amanda.toney@ dhha.org	Amanda Greene Toney, MD	amanda.toney@ dhha.org	No	https://www.denverultrasound.org/fellowship
Emory University, Atlanta, GA	Gregg Helland, MD	gregg.helland@ emory.edu	E. Liang Liu, MD Lekha Shah, MD	emberlynn.liang.liu@emory.edu lashah@ emory.edu	Yes	https://med.emory.edu/departments/emergency-medicine/education/fellowship/ultrasound/index.html
Icahn School of Medicine at Mount Sinai-Peds, New York, NY	Bret Nelson, MD Jim Tsung, MD	bret.nelson@mssm.edu james.tsung@mountsinai.org	Jim Tsung, MD	james.tsung@ mountsinai.org	Yes	https://www.eusfellowships.com/programs/details?id=33
Indiana University - Riley Hospital for Children, Indianapolis, IN	Benjamin Nti, MD	bnti@iu.edu	Benjamin Nti, MD	bnti@iu.edu	No	https://medicine.iu.edu/emergency-medicine/education/fellowship/ultrasound
John’s Hopkins Children’s Center, Baltimore, MD	J. Kate Deanehan, MD	jdeaneh1@jh.edu	J. Kate Deanehan, MD	jdeaneh1@jh.edu	No	https://www.hopkinsmedicine.org/johns-hopkins-childrens-center/what-we-treat/specialties/emergency-medicine
Maimonides Medical Center, Brooklyn, NY	Lawrence Haines, MD, MPH	Ihaines@ maimonidesmed. org	Leily Naraghi, MD	lnaraghi@ maimonidesmed.org	Yes	https://www.maimonidesem.org/fellowship/ultrasound
Medical University of South Carolina, Charleston, SC	Ryan M. Barnes, DO	barnesry@ musc.edu	Aalap Shah, MD	shahaa@ musc.edu	Yes	https://www.eusfellowships.com/programs/details?id=100
Morgan Stanley Children’s Hospital, Columbia University, New York, NY	Lorraine Ng, MD	ln2136@cumc.columbia.edu	Joni Rabiner, MD	jer2212@cumc.columbia.edu	No	https://www.emergencymedicine.columbia.edu/education/fellowships/pediatric-emergency-ultrasound-fellowship
Nationwide Children’s Hospital/Ohio State University, Columbus, OH	Creagh Boulger, MD	creagh.boulger@ osumc.edu	Delia Gold, MD	delia.gold@nationwidechildrens.org	No	https://www.eusfellowships.com/programs/details?id=36
New York Presbyterian Brooklyn Methodist Hospital, Brooklyn, NY	Sharon Yellin, MD	shy9015@nyp. org	Raymond Chen, DO	rmc9019@nyp.org	Yes	https://eusfellowships.com/programs/details?id=18
Norton Children’s Hospital, Louisville, KY	Fred Warkentine, MD	fred.warkentine@louisville.edu	Rebecca Starr, DO	rebecca.starr@louisville.edu	No	https://www.eusfellowships.com/programs/details?id=176
Sunderland Royal Hospital, Sunderland, United Kingdom	David McCreary, MBBS	david.mccreary2@nhs.net	David McCreary, MBBS	david.mccreary2@nhs.net	No	https://www.sunderlandultrasoundsociety.com/
The Hospital for Sick Children, Toronto, ON, Canada	Gregory Harvey, MD	gregory.harvey@ sickkids.ca	Maya Capua, MD	c/o: kelly.sobie@ sickkids.ca	No	https://p2sk.ca/
UCSD/Rady Children’s Hospital, San Diego, CA	Atim Ekpenyong MD	auya@ucsd.edu	Mylinh Nguyen MD	mnguyen12@ rchsd.org	No	https://pediatrics.ucsd.edu/divisions/emergency-medicine/fellowship-education/fellowship-overview.html
University of Arkansas, Little Rock, AR	Gregory Snead, MD	grsnead@uams.edu	Jason Arthur, MD	jarthur@ uams.edu	Yes	https://medicine.uams.edu/emergencymedicine/divisions/ultrasound/
UCSF Benioff Children’s Hospital, San Francisco, CA	Aaron Kornblith, MD	aaron.kornblith@ucsf.edu	Margaret Lin-Martore, MD Aaron Kornblith, MD	margaret.lin-martore@ucsf. edu aaron.kornblith@ ucsf.edu	No	https://emergency.ucsf.edu/pediatric-emergency-ultrasound-fellowship
University of Massachusetts, Worcester, MA	Timothy Gleeson, MD	timothy.gleeson@umassmemorial.org	Robert Lindsay, MD Zachary Binder, MD	robert.lindsay@umassmemorial.org zachary.binder@umassmemorial.org	Yes	https://www.umassmed.edu/emed/education/fellowship/ultrasound/
University of Michigan C.S. Mott Children’s Hospital, Ann Arbor, MI	Nik Theyyunni, MD	ntheyyu@med. umich.edu	Nicole Klekowski, MD David Haidar, MD	nklekows@med.umich.edu dahaidar@med.umich.edu	Yes	https://medicine.umich.edu/dept/emergency-medicine/education/fellowships/advanced-emergency-medicine-ultrasonography-fellowship
UPMC Children’s Hospital of Pittsburgh, Pittsburgh, PA	Desiree Noel Wagner Neville, MD	desiree.neville@upmc.edu	Vipin Philip, MD	philipv@upmc.edu	No	https://www.pediatrics.pitt.edu/divisions/emergency-medicine/education-and-training
Yale University, New Haven, CT	Chris Moore, MD	chris.moore@yale.edu	Antonio Riera, MD	antonio.riera@yale.edu	Yes	https://medicine.yale.edu/pediatrics/education/fellowships/emergency-med/pocus-fellowship/

We extend a special thanks to the POCUS Journal for agreeing to publish the listing of this important and expanding area in the field of Pediatric Emergency Medicine. 
